# Distinct leaf functional traits of *Tamarix chinensis* at different habitats in the hinterland of the Taklimakan desert

**DOI:** 10.3389/fpls.2022.1094049

**Published:** 2023-01-18

**Authors:** Mawlida Tayir, Yue Dai, Qingdong Shi, Anwar Abdureyim, Flora Erkin, Wanyuan Huang

**Affiliations:** ^1^ College of Ecology and Environment, Xinjiang University, Urumqi, China; ^2^ Key Laboratory of Oasis Ecology, Xinjiang University, Urumqi, China; ^3^ Key Laboratory of Smart City and Environment Modelling of Higher Education Institute, Xinjiang University, Urumqi, China; ^4^ College of Geography and Remote Sensing Science, Xinjiang University, Urumqi, China

**Keywords:** Oasis, abiotic factors, biological factors, groundwater depth, slow-investment strategy

## Abstract

Leaf functional traits reflect plant adaptive strategies towards environmental heterogeneity. However, which factor play the key role of plasticity of leaf functional traits among various variable environmental factors remains unclear in desert hinterland oasis area. Here, we analyzed variations in leaf water content (LWC), *δ* ^13^C values of leaves (*δ* ^13^C), specific leaf area (SLA), leaf organic carbon concentration (LOC), leaf total nitrogen concentration (LTN), leaf total phosphorus concentration (LTP), and leaf C: N: P stoichiometry in *Tamarix chinensis* growing in five habitats at the Daliyabuyi, a natural pristine oasis in northwestern China, that differ abiotically and biotically. The spatial heterogeneity of leaf functional traits was evident. Abiotic factors vitally influence leaf functional traits, of which groundwater depth (GWD) and soil C: N stoichiometry (SOC: STN) are crucial. GWD exhibited close relationships with LWC (*P* < 0.05) and LOC: LTP (*P* < 0.01), but not *δ* ^13^C. Soil water content (SWC) and SOC: STN were negatively related to SLA (*P* < 0.01; *P* < 0.05). While, SOC: STN showed positive relationships with LOC: LTN (*P* < 0.05). As for biological factors, we found *T. chinensis* in habitat with *Sophora alopecuroidies* had the highest LTN, possibly as a result of N fixation of leguminous plants (*S. alopecuroidies*) promotes the N concentration of *T. chinensis*. Close relationships also existed between leaf functional traits, LWC showed significantly negatively relatd to *δ* ^13^C, LOC: LTN and LOC: LTP (*P* < 0.05), whereas *δ* ^13^C had positively correlated with LOC: LTN (*P* < 0.01) but negatively correlated with LTN (*P* < 0.05). *T. chinensis* had relative higher LWC couple with lower *δ* ^13^C, and exhibiting lower C, N, P in leaves and their stoichiometric ratios, and also lower SLA which compared with other terrestrial plant. Such coordinations suggesting that *T. chinensis* develops a suite of trait combinations mainly tends to more conservative to response local habitats in Daliyabuyi, which is contribute to understand desert plant resource acquisition and utilization mechanisms in extremely arid and barren environments.

## 1 Introduction

Plant functional traits indicate the ability of plants to adapt to environmental changes (Perez-Harguindeguy et al., 2013). Leaves are the organs that experience the most exposure to the atmospheric environment. Leaf functional traits are therefore a significant part of plant functional traits, which are firmly relevant to resource acquisition and utilization, as well as plant biomass (Zirbel et al., 2017). Leaf functional traits variation across habitats has long interested ecologists, clarifying the key factors which influence the variation makes efforts to understand how plants response to evolutionary history and global environmental changes (Díaz et al., 2004; Poorter and Bongers, 2006; Hernández-Calderón et al., 2014; An et al., 2021). Soil properties are considered as key factors since they are crucial for plant growth, composition, and establishment. In earlier study, Han et al. (2005) suggested a positive correlation between soil phosphorus (P) concentration and leaf P concentration in their investigation across 753 terrestrial plant species in China. Previous studies have also found that soil water content (SWC) is related to leaf functional traits such as leaf water content (LWC), specific leaf area (SLA) and leaf P concentrations, certainly, for most plants with deep roots in desert, deep soil water is the key to regulate their various leaf traits (Akram et al., 2020; Li et al., 2021). In general, the optimum groundwater depth (GWD) is more favorable to the growth of desert plants, previous studies have revealed a close relationship between leaf functional traits, e. g. LWC, *δ* ^13^C values of leaves (*δ* ^13^C) as well as leaf element concentrations, and GWD both in the lower reaches of the Tarim River and the southern rim of the Taklamakan Desert (Chen et al., 2013; Zhang et al., 2018). In addition to abiotic factors (environmental factors), biological factors (community composition) also make contributions on leaf functional traits variation. Substantial evidence exists that for symbiotic leguminous and non-leguminous species, nitrogen (N) fixation affects leaf carbon (C), N and P concentrations, as well as leaf C: N: P stoichiometry of non-leguminous species (Nyfeler et al., 2009; Zeng et al., 2010; Zhang et al., 2016) *via* its impacts on soil chemical and physical properties, which clarified the phenomena and factors driving the correlation between differential community composition and leaf elemental concentrations and stoichiometric characteristics. Moreover, previous studies have also revealed significant species diversity effects on leaf elemental concentrations (van Ruijven and Berendse, 2005; Abbas et al., 2013). In summary, both abiotic and biological factors are believed to be the primarily reason for community assembly, which results in leaf functional traits variation in response to habitat spatial heterogeneity.

Despite leaf functional traits are widely variable across distinct habitats, they don’t vary indepently but connected (Reich and Oleksyn, 2004; Wright et al., 2004a; An et al., 2021). For the past nearly two decades, since the well-known ‘leaf economics spectrum’ (LES) highlights a trade-off from the quick to the slow return on investments of nutrients and dry mass of leaves, numerous previous studies have demonstrated strong relationships among distinct leaf traits at individual and community level by investing to regional-sacle and global-scale, and the correlations between key leaf traits taking more concern by ecologists, which can help to better describe the plant adaptation to the environment (McGroddy et al., 2004; Reich and Oleksyn, 2004; Wright et al., 2004a; Wright et al., 2004b; Easlon et al., 2014). Take leaf physiological traits for an example, several studies have revealed that simultaneous measurement of both LWC and *δ* ^13^C provides a better understanding water use efficiency (WUE) response towards a reducing water supply in native species growing in arid or semi-arid environments (Chen et al., 2007; Bouda et al., 2013; Easlon et al., 2014). Several studies have reported that species with higher SLA always pair with higher leaf N and P concentrations, these acquisitive traits result in a faster return on resource investment and promote rapid plant growth (Wright and Cannon, 2001; Wright et al., 2004b; Kang et al., 2021). On the contrary, the slow return on investments and growth rate result from more conservative traits combinations, for example, lower SLA with lower leaf elements traits and their stoichiometric characteristics (Kang et al., 2021). Accordingly, exploring the trait-trait relationships provide a better understanding of how plants develop a suite of trait combinations that enable them to adapt to native habitats.

Old-World genus *Tamarix* belongs to Tamaricaceae family has about 54 species of shrubs and trees, and are grown in dry, salty, or riparian conditions (Gaskin and Schaal, 2002)*. Tamarix* is one of the main components in desert oases and plays a key role in protecting oases and maintaining their stability (Xun and Wang, 2015). *Tamarix chinensis* is a species of *Tamarix*, as a typical dominanting vegetation in the hinterland of the Taklimakan desert, Daliyabuyi (Li et al., 2021; Wan et al., 2022). Recent studies on LWC and *δ* ^13^C related to water usage, have concluded that *Tamarix* usually adopts a conservative water-use strategy under drought and salty stress (Marhaba et al., 2020; Hussain et al., 2022). Sun et al. (2017) measured C, N, and P concentrations and their stoichiometric ratios in samples of four species of *Tamarix* from 30 sites in eight different Chinese provinces. They have demonstrated, at a large scale, that *Tamarix* species growing in arid conditions had lower C and higher N and P in leaves, and variation in leaf stoichiometric compositions are mainly drived by climatic factors and less influenced by geographical changes and soil nutrients. In contrast, another study in northeastern Spain (Camarero, 2019), the author suggested that leaf functional traits of *Tamarix* were less responsive to climate variability. Although previous studies have summarized the response of leaf functional traits of *Tamarix* species to drought and salinity stress on a large scale, and the comparison of leaf functional traits between *Tamarix* species and other species is relatively common (Rong et al., 2014; Sun et al., 2017; Camarero, 2019; Marhaba et al., 2020; Hussain et al., 2022). But here’s another fact that the discussion of habitat spatial heterogeneity was limited, and the possible biological factors were less considered. Given the fact that different traits may respond differently to the same stress and that plants may select different foliar trait-trait combinations to adapt to various stresses (Messier et al., 2010), it is important to understand the main determinants of the variation in the leaf functional traits of *T. chinensis*, and the interrelation in each leaf functional traits in extremely arid and barren environments.

With these aims in mind, we focused on nine leaf functional traits of *T. chinensis* in the hinterland of the Taklimakan desert, Daliyabuyi, that were collected from five different habitats with different vegetation communities, trying to address the following questions: (i) how do leaf functional traits of *T. chinensis* differ at different habitats? (ii) what are the main factors in such leaf functional traits variation? and (iii) how do *T. chinensis* leaf functional traits interrelate?

## 2 Materials and methods

### 2.1 Study area

**Figure 1:**
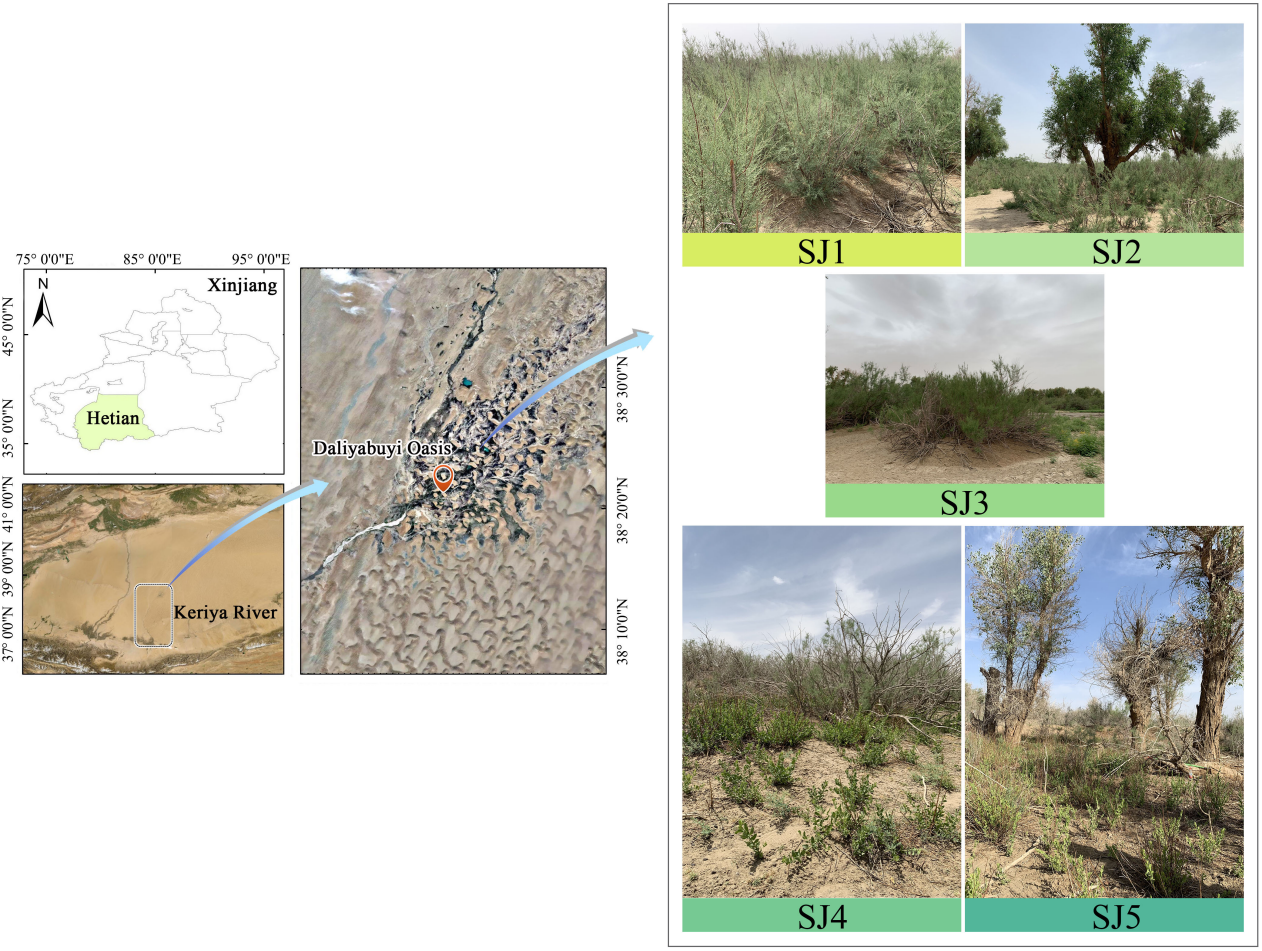
Study area location in Xinjiang, China. The abbreviations of the different habitats are as followed: *T. chinensis* population (SJ1), *T. chinensis - P. euphratica* (SJ2), *T. chinensis - S. alopecuroidies* (SJ3), *T. chinensis - K. caspia - A. sparsifolia* (SJ4), *T. chinensis - K. caspia - A. sparsifolia - P. euphratica* (SJ5).

Daliyabuyi oasis (38° 16′ – 38° 37′ N, 81° 05 ′ – 81° 46′ E), is lotcated in Yutian County, Xinjiang, China (**Figure 1**). The study site has a typical warm temperate, arid desert climate (Wan et al., 2022). From the Yu Tian meteorological observatory in this study area, it has a mean air temperature of 11.6°C for the year, an average precipitation of fewer than 10 mm. This oasis still retains its ‘pristine’ state and has experienced minimal human interference; therefore, it currently thrives under natural conditions (Shi et al., 2021). *Populus euphratica* and *T. chinensis* are the dominant plant species in this community. Other common plant species are *Apocynum ventum*, *Phragmites australis*, *Karelinia caspia*, *Alhagi sparsifolia*, *Sophora alopecuroidies*, and *Achnatherum splendens*. The groundwater depth of the oasis ranges between 2 – 9 m from south to north (Imin et al., 2021; Wan et al., 2022).

### 2.2 Experimental design

In June 2021, we selected five sampling sites near already established groundwater observation wells within the Daliyabuyi oasis; these wells are secluded and far away from villages; thus, they have experienced little man-made disturbance. These five sampling sites were composed of different vegetation communities, namely: *T. chinensis* population (SJ1), *T. chinensis* -* P. euphratica* (SJ2), *T. chinensis* - *S. alopecuroidies* (SJ3), *T. chinensis* -* K. caspia* -* A. sparsifolia* (SJ4), *T. chinensis* -* K. caspia* -* A. sparsifolia* -* P. euphratica* (SJ5). Three 10 m × 10 m sample plots were established in each sampling site according to vegetation growth and groundwater level. Specifically, we recorded *T. chinensis* plant height and canopy width, sample location, and groundwater depth (**Table 1**).

**Table 1:** Basic characteristics of *T. chinensis* in each of the different habitats, together with relevant environmental factors.

	SJ1	SJ2	SJ3	SJ4	SJ5
Sample location	38°33′52.9′′N 81°90′59.7′′E	38°33′52.9′′N 81°90′59.7′′E	38°33′52.9′′N 81°90′59.7′′E	38°33′57.4′′N 81°93′24.5′′E	38°33′57.4′′N 81°93′24.5′′E
Height/m	1.50 ± 0.35	1.32 ± 0.30	1.72 ± 0.31	1.73 ± 0.28	1.84 ± 0.54
Canopy width/m	0.95 ± 0.16	1.01 ± 0.26	2.18 ± 0.52	1.45 ± 0.49	1.16 ± 0.32
Groundwater depth/m	3.00	3.00	2.40	1.80	1.80

**Figure 2:**
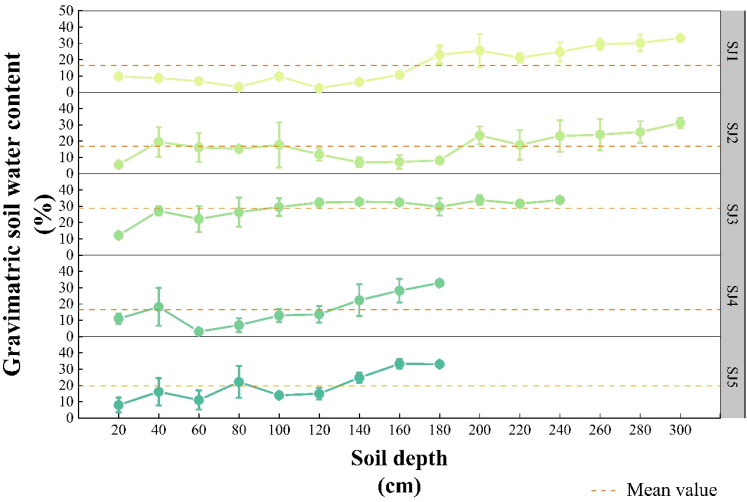
Vertical soil water content profiles of the different habitat conditions in the Daliyabuyi oasis.

Values represent means ± SE. The abbreviations of the different habitats are the same as in **Figure 2**.

Our study selected nine ecologically important and closely related leaf functional traits, the details of which can be obtained in **Table 2**.

**Table 2:** List of leaf functional traits and their ecological significance.

Traits	Abbreviations	Unit	Ecological significance and references
Leaf water content	LWC	%	Reflecting stress tolerance and water use strategy (Chen et al., 2007)
*δ* ^13^C values of leaves	*δ* ^13^C	‰	As a tool to measure water-use efficiency (Farquhar and Richards, 1984; Dawson et al., 2002)
Specific leaf area	SLA	cm^2^·g^-1^	Reflecting growth rate and resource acquisition (Wright et al., 2004a; Reich, 2014)
Leaf organic carbon concentration	LOC	g·kg^-1^	Explanation of elemental composition and plant structure (Aerts and Chapin, 2000; Reich and Oleksyn, 2004)
Leaf total nitrogen concentration	LTN	g·kg^-1^	
Leaf total phosphorus concentration	LTP	g·kg^-1^	
C: N ratio	LOC: LTN	/	Explanation of ecological composition and interactions of organisms with the surrounding environment (Elser et al., 2000b; Elser et al., 2010)
C: P ratio	LOC: LTP	/	
N: P ratio	LTN: LTP	/	

### 2.3 Sampling and measurements

To analyze leaf functional traits, fully expanded and sun exposed leaves from three to four healthy and mature *T. chinensis* plants were randomly collected in each plot. We first collected leaf samples to determine LWC and *δ* ^13^C. Specifically, fresh leaves were first weighed, then dried at 75°C to a consistent mass, and weighed again to estimate LWC. In the laboratory, leaves were washed and subsequently fixed at 105°C, oven dried at 75°C, and then pulverized manually using a mortar and pestle. Ground samples were weighed and labeled, and then kept in plastic bags. *δ* ^13^C of *T. chinensis* leaves were calculated with a Thermo Delta V Advantage (DELTA-V; Thermo Fisher Scientific, Waltham, MA, USA). Equation (1) was used to compute the carbon isotope abundance (*δ* ^13^C, ‰), which was represented as the sample’s isotopic ratio in relation to the Pee Dee Belemnite (PDB) standard (Qin et al., 2020):


(1) $$\delta^{13}\text{C}=\left(\frac{{\text{ R}}_{\text{sample}}}{{\text{R}}_{\text{standard}}}\;-1\right)\times 1000‰$$

A second collection was made for measuring SLA, LOC, LTN, LTP, and C: N: P. Within 48 hours, leaves were laid out next to a ruler on a white background and photographed. Through the use of Adobe Photoshop 7.0, the average leaf area per specimen was able to calculate. All leaves were then dried in an oven at 75°C before being weighed to establish their dry mass. Equation (2) was then used to calculate SLA:


(2) $$\text{SLA}=\frac{\text{leaf area }\left({\text{cm}}^{2}\right)}{\text{dry weight }\left(\text{g}\right)}$$

Finely ground dried leaves with a mill subsequently passed through an 80-mesh screen. This was then used to measure LOC (K_2_MnO_4_ volume method), LTN (Kjeldahl acid-digestion method), LTP (molybdenum-antimony solution was reacted with colorimetrically at 700 nm). LOC, LTN, and LTP were used to calculate leaf C: N: P stoichiometry.

Three points were selected near each *T. chinensis* sample for soil sample collection. Soils were sampled in 20 cm intervals, using a soil drill, until the soil drill reached the groundwater table. In order to measure the SWC, soil samples were put in aluminum boxes. In each plot, surface soil samples were taken 0 – 20 cm below the plant canopy. Each soil sample was separated into two subsamples after being put through a 2 mm screen. After being air dried, ground in a mill, and passed through a 60-mesh screen, the first subsample was analyzed for SOC (K_2_MnO_4_ volume method), STN (Kjeldahl acid-digestion method), and STP (HCLO_4_-H_2_SO_4_ method). SOC, STN, and STP were used to calculate the C: N: P stoichiometry of soil, while the second subsample was used to analyze soil pH.

### 2.4 Data analysis

SPSS 22.0 was used for all data analysis and all data are represented as means ± SE. Prior to analysis, all data were examined for normality and homoscedasticity. All graphs were plotted with Origin 2021. To statistically analyzed abiotic factors’ and traits’ comparison across five distinct habitats, one-way ANOVA was performed using SPSS 22.0. To test the significance among abiotic factors classes and traits classes, *post hoc* multiple comparisons were based on the Least Significant Difference (LSD) method at adjusted 0.05 significance level. To demonstrate the main abiotic factors were correlated with selected leaf functional traits in five different habitats and to test the correlations among traits selected, Pearson correlation analysis were performed.

## 3 Results

### 3.1 Soil parameter variation in different habitats

In note, SWC from five distinct habitats did not significantly differ from one another (except SJ3), and these results are reported only to reveal general tendencies for a comprehensive discussion. SWC in the five habitats initially fluctuated slightly, after which it increased rapidly (**Figure 2**). Specifically, SWC of SJ3 was significantly higher than the other four habitats (SJ1, SJ2, SJ4, SJ5) (*P* < 0.05), with a mean value of 28.65 %. The mean values of SWC in SJ1, SJ2, SJ4, SJ5 were as fowllowed respectively: 16.34 %, 16.89 %, 16.59 %, 19.01 %.

Soil C, N, P, C: N, C: P, and N: P ratios, as well as pH, differed significantly among the habitats (**Table 3**, *P* < 0.05). SOC, STN, and STP ranged between 2.57 – 5.15 g·kg^-1^, 0.097 – 0.192 g·kg^-1^, and 0.51 – 0.61 g·kg^-1^, respectively. Furthermore, SOC, STN, and STP in SJ1 were higher compared to the other habitats. The SOC of SJ1 and SJ2 were significantly higher than in SJ3 and SJ4. Also, the STN of SJ1 and SJ2 were significantly higher than in SJ3, SJ4, and SJ5, and the STP of SJ1 was significantly higher than in SJ3, SJ4, and SJ5. SOC: STN, SOC: STP, and STN: STP ranged between 24.92 – 29.36, 4.67 – 8.54, and 0.18 – 0.33, respectively. SOC: STN of SJ4 and SJ5 were significantly higher than in SJ1, SJ2, and SJ3. SOC: STP of SJ1 and SJ2 were significantly higher than in SJ3. STN: STP of SJ2 was significantly higher than in SJ3. Finally, soil pH ranged between 8.49 – 9.15, and the pH of SJ4 was significantly higher than in SJ1 and SJ5.

**Table 3:** Soil pH, C, N, P, and stoichiometric characteristics (C: N, C: P, N: P) among different habitats in the Daliyabuyi oasis.

	SJ1	SJ2	SJ3	SJ4	SJ5
pH	8.49 ± 0.12^c^	8.93 ± 0.18^ab^	8.98 ± 0.13^ab^	9.15 ± 0.25^a^	8.80 ± 0.13^b^
SOC (g·kg^-1^)	5.15 ± 0.7^4a^	4.77 ± 1.6^4a^	2.57 ± 0.17^c^	3.09 ± 0.24^bc^	3.58 ± 0.42^abc^
STN (g·kg^-1^)	0.192 ± 0.015^a^	0.191 ± 0.067^a^	0.097 ± 0.006^b^	0.105 ± 0.009^b^	0.122 ± 0.016^b^
STP (g·kg^-1^)	0.61 ± 0.03^a^	0.58 ± 0.02^ab^	0.55 ± 0.01^bc^	0.51 ± 0.01^c^	0.51 ± 0.03^c^
SOC: STN	26.73 ± 1.93^bc^	24.92 ± 1.29^c^	26.40 ± 0.40^bc^	29.36 ± 0.91^a^	29.35 ± 0.45^a^
SOC: STP	8.54 ± 1.56^a^	8.25 ± 2.96^a^	4.67 ± 0.32^b^	6.05 ± 0.57^ab^	7.02 ± 0.51^ab^
STN: STP	0.32 ± 0.04^ab^	0.33 ± 0.12^a^	0.18 ± 0.01^c^	0.21 ± 0.02^bc^	0.24 ± 0.01^abc^

Different lowercase letters indicate significant differences among the habitats (P < 0.05). The abbreviations of the different habitats and soil parameters are the same as in **Figures 1** and **Figure 4**, respectively.

### 3.2 Leaf functional trait variation in different habitats

**Figure 3:**
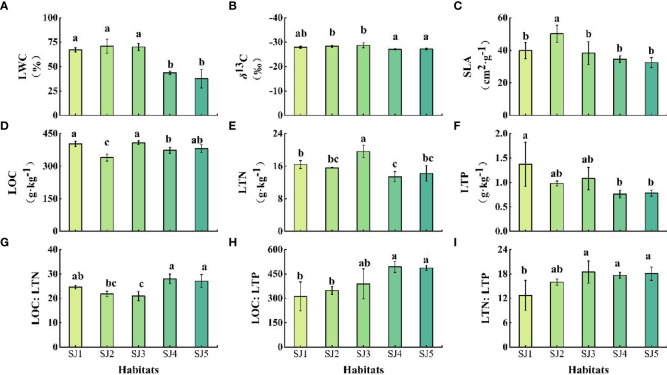
Vertical soil water content profiles of the different habitat conditions in the Daliyabuyi oasis. Note: Different lowercase letters indicate significant differences among the habitats (P < 0.05). Error bars indicate standard errors of the mean for triplicate measurements. The abbreviations of the leaf functional traits and different habitats are the same as in **Table 2** and **Figure 1**, respectively. **(A)** LWC; **(B)** δ13C; **(C)** SLA; **(D)** LOC; **(E)** LTN; **(F)** LTP; **(G)** LOC: LTN; **(H)** LOC: LTP; **(I)** LTN: LTP.

*T. chinensis* leaf functional traits differed significantly between the different habitats (**Figure 3**, *P* < 0.05). Specifically, LWC ranged between 37.61 – 70.8 %, and was significantly higher in SJ1, SJ2, and SJ3 compared to SJ4 and SJ5 (**Figure 3A**). *δ* ^13^C ranged between -28.70 – -27.08 ‰, and was significantly higher in SJ4 and SJ5 compared to SJ2 and SJ3 (**Figure 3B**). SLA ranged between 32.45 cm^2^·g^-1^ – 50.19 cm^2^·g^-1^, and was significantly higher in SJ2 compared to other habitats. In contrast, there was no significant difference in SLA among SJ1, SJ3, SJ4, and SJ5 (**Figure 3C**). LOC, LTN, and LTP ranged between 339.60 – 408.07 g·kg^-1^, 13.41 – 19.61 g·kg^-1^, and 0.76 – 1.37 g·kg^-1^, respectively (**Figures 3D-F**). LOC was significantly higher in SJ1 and SJ3 compared to SJ2, SJ4, and SJ5. LTN was significantly higher in SJ3 compared to the other habitats. LTP was significantly higher in SJ1 compared to SJ4 and SJ5. LOC: LTN, LOC: LTP, and LTN: LTP ranged between 24.6 – 27.95, 312.23 – 493.33, and 12.72 – 18.46, respectively (**Figures 3G-I**). LOC: LTN was significantly higher in SJ4 and SJ5 compared to SJ3. LOC: LTP was significantly higher in SJ4 and SJ5 compared to SJ1 and SJ2. LTN: LTP was significantly higher in SJ3, SJ4, and SJ5 compared to SJ1.

### 3.3 Correlations between leaf functional traits and environmental factors

**Figure 4:**
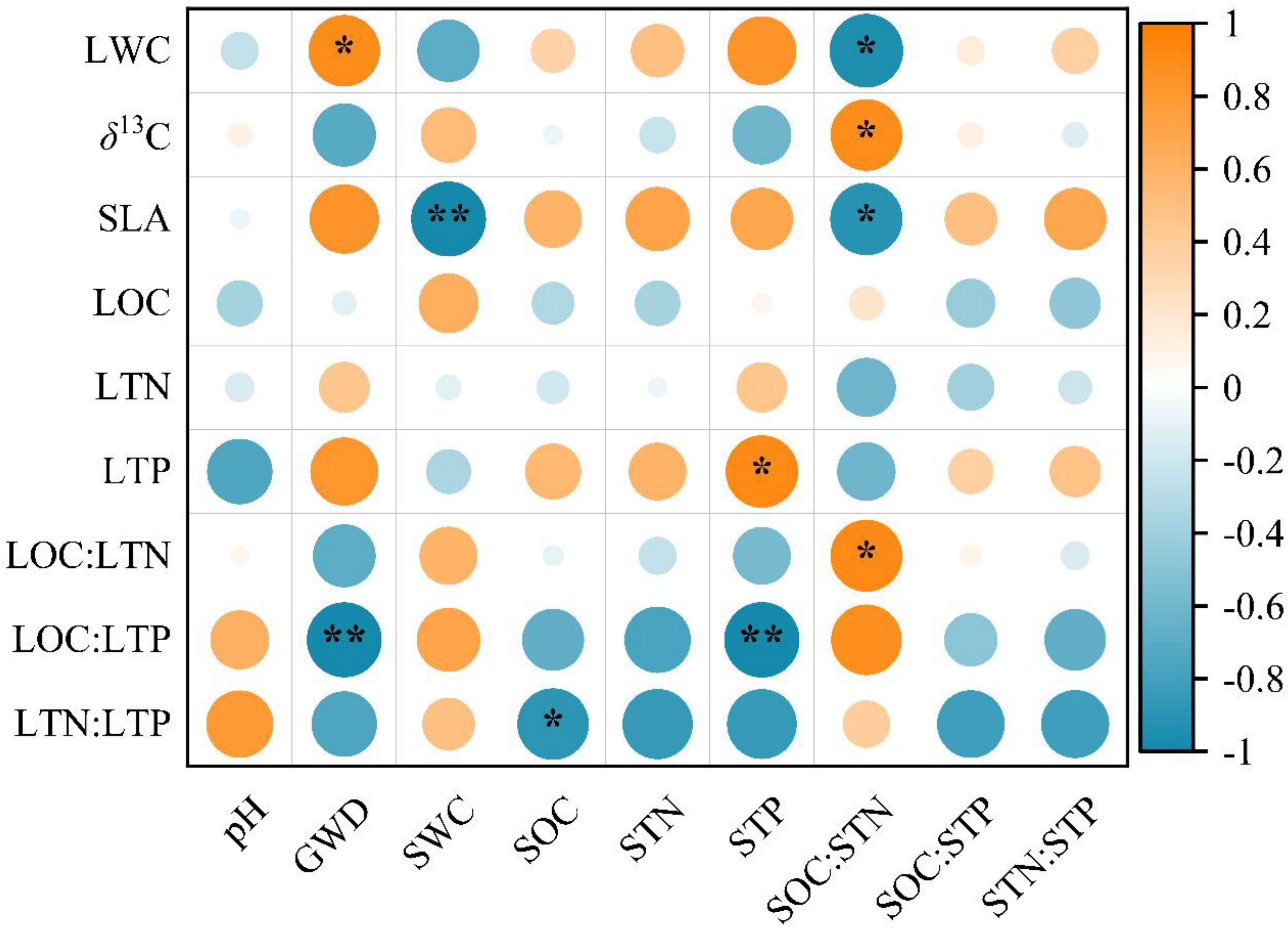
Correlations between *T. chinensis* leaf functional traits and environmental factors. The orange circle represents a positive correlation, and the blue circle represents a negative correlation. Correlations are significant at ** *P* < 0.01 and * *P* < 0.05. The abbreviations of the leaf functional traits are the same as in **Table 2**. The abbreviations of the abiotic variables are as follows: Soil pH (pH), soil water content (SWC), soil organic carbon concentration (SOC), soil total nitrogen concentration (STN), soil total phosphorus concentration (STP), soil C: N: P stoichiometry (SOC: STN, SOC: STP, STN: STP).

GWD, SWC, SOC, STP, and SOC : STN traits were correlated with specific leaf functional traits studied, but not wiht LOC and LTN (**Figure 4**). Both LWC and SLA were significantly and negatively correlated with SOC: STN (*P <* 0.01), while *δ* ^13^C and LOC: LTN were significantly and positively correlated with SOC: STN (*P* < 0.05). Furthermore, LWC was significantly and positively correlated with GWD (*P* < 0.05), and SLA was significantly and negatively correlated with SWC at a 180 cm depth (*P* < 0.01). Additionally, LTP was significantly and positively correlated with STP (*P* < 0.05), while LOC: LTP was significantly and negatively correlated with STP and GWD (*P* < 0.01). Finally, LTN: LTP was significantly and negatively correlated with SOC (*P* < 0.05).

### 3.4 Correlations between leaf functional traits

**Figure 5:**
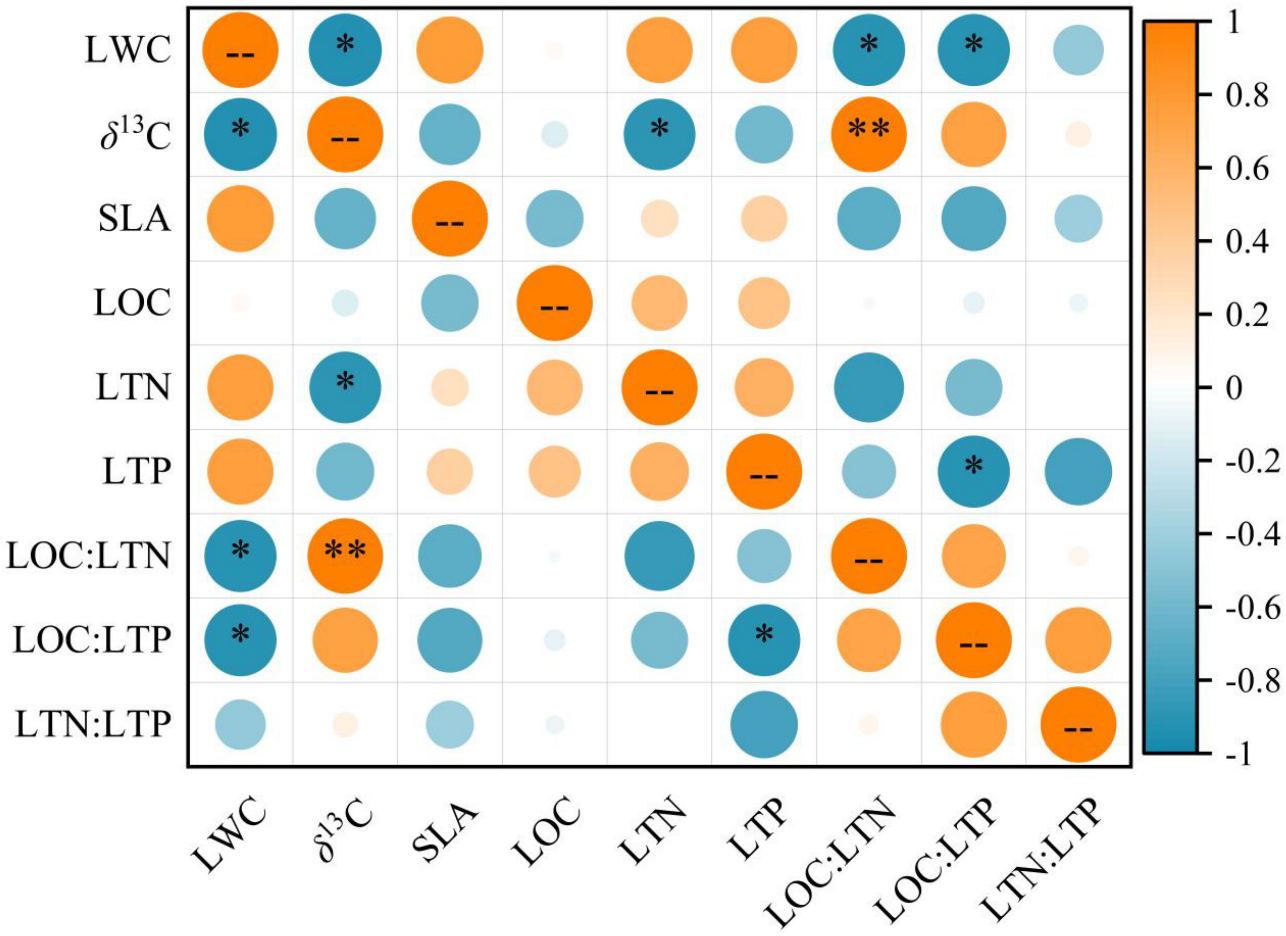
Correlations between *T. chinensis* leaf functional traits. The orange circle represents a positive correlation, and the blue circle represents a negative correlation. Correlations are significant at ** *P* < 0.01 and * *P* < 0.05. The abbreviations of the leaf functional traits are the same as in **Table 2**.

Pearson correlation result. yielded different correlations for the leaf functional traits (**Figure 5**). LWC exhibited a significant negative correlation with LOC: LTN and LOC: LTP (*P* < 0.05). *δ* ^13^C was significantly and negatively correlated with LWC and LTN (*P* < 0.05), and significantly and positively correlated with LOC: LTN (*P* < 0.01). Moreover, LTP was significantly and negatively correlated with LOC: LTP (*P* < 0.05), however, considering the inevitable autocorrelation between these two values, the relationship is not analyzed in detail.

## 4 Discussion

### 4.1 Correlations of abiotic and biological variables with leaf functional traits

Our comparative study of *T. chinensis* leaf functional traits in different habitats found that habitat spatial heterogeneity resulted in leaf functional trait differences. This is caused by both abiotic and biological factors, among which abiotic factors showed correlation with leaf functional traits (**Figure 4**). In this study, GWD of the five habitats (SJ1 – SJ5) we selected varied between 1.80 – 3.00 m, healthy and mature *T. chinensis* individuals, growing in alkaline soils with pH ranging from 8.49 – 9.15 (**Tables 1**, **3**). Comparison of leaf functional traits and in these five habitats, and the main factors for such differences are described below.

First, LWC ranged between 37.61 – 70.81 %, while *δ* ^13^C ranged between -28.70 – -27.08 ‰ (**Figures 3A, B**). The ranges of these two traits variation we obtained is basically consistent with the results of previous studies on *T. chinensis* (Xia et al., 2016; Imin et al., 2021; Wan et al., 2022). According to the result of Pearson correlation result. (**Figure 4**), positive relationships existed between GWD and LWC, considering that a GWD range of 2 – 6 m is optimal for constructive species such as *Tamarix* in desert area (Chen et al., 2013), besides, mature *T. chinensis* have a strong water absorption capacity since they can access deep underground water sources (Dong et al., 2020), it is therefore understandable that LWC of *T. chinensis* in the habitats (SJ1, SJ2 and SJ3) at GWD within 2.4 – 3.0 m are significantly higher than that of SJ3 and SJ4 growing in GWD of 1.8 m in our study. Secondary soil salinization had an impact on the shallow GWD (Chen et al., 2013), which might be the main factor limiting development of *T. chinensis* at GWD of 1.8 m. We also compared *T. chinensis* foliar *δ* ^13^C values among the five habitats. However, the cause of *δ* ^13^C differences was not reflected in GWD changes, similar to the findings of Marhaba et al. (2020) in previous work, but different from the results of Imin et al. (2021) conducted that *T. chinensis* foliar *δ* ^13^C values response differently to GWD, within the range of 2.1 – 4.3 m, in Daliyabuyi oasis. Foliar *δ* ^13^C values reflects WUE and is positively correlated with WUE (Farquhar and Richards, 1984). The uncorrelated results may be due to the case that *T. chinensis* retains a relatively high transpiration rate even when the water-absorbing layer is deep, so as to avoid strong drought stress and maintain a stable WUE (Wang et al., 2017). Since both photosynthesis and transpiration contribute to WUE (Bierhuizen and Slatyer, 1965), factors that influence either of these physiological processes are likely related to WUE; On the other hand, nutrients enhance photosynthetic rate while having little impact on transpiration rate (Patterson et al., 1997), leading to an increase in WUE. The significant positive relationship between SOC: STN (*P* < 0.05) (**Figure 4**), which is related to soil nutrient conditions, and *δ* ^13^C in our study, providing convincing evidence for the above views.

Second, SLA values ranged between 32.45 – 50.19 cm^2^·g^-1^ (**Figure 3C**), the mean of which was 39.06 ± 6.87 cm^2^·g^-1^. This result was lower (53 ± 2 cm^2^·g^-1^) compared to *Tamarix*, which is Mediterranean plant genus growing in north-eastern Spain (Camarero, 2019). This comparison implies that species in habitats with abundant resources typically have higher SLA values than species in conditions with scarce resources (Cornelissen et al., 2003). In our study, SLA showed a significantly negative correlated with both SWC (*P* < 0.01) and SOC: STN (*P* < 0.05) (**Figure 4**). Our assumptions may overstate the range of SWC changes in the five habitats in our study because we expected that SLA declines with increasing aridity and nutrient shortages in native habitats (Fonseca et al., 2000; Wright and Cannon, 2001). On the one hand, the mean value of SJ3 was significantly higher than that of the other four habitats, and there was no statistical difference among these four habitats (**Figure 2**). According to Wan et al. (2022) investigation in Daliyabuyi, mature *T. chinensis* main water supply is in the deep soil water between 140 and 300 cm. The SWC of SJ3 at 140 – 300 cm in our research is around 30 %, which is higher than the SWC of the deep soil in previous studies (Marhaba et al., 2020; Wan et al., 2022). This might be as a result of river overflow in the habitat before to sampling. Short-term waterlogging stress prevents the roots’ access to oxygen (Yang et al., 2011), thus inhibiting plant growth. Waterlogging results in some changes in leaf functional traits, such as the reduction of SLA (Zuniga-Feest et al., 2017). On the other hand, our experiments were restricted to only one season and the majority of changes in SLA are likely to be driven by soil, it is difficult to separate the contributions of the various factors. As a result, future research on the long-term effects of SLA and other abiotic factors is necessary.

Third, the average LOC, LTN, and LTP of *T. chinensis* in the five habitats were 380.97 ± 27.97 g·kg^-1^, 15.84 ± 2.49 g·kg^-1^, and 1.00 ± 0.30 g·kg^-1^, respectively, and were lower compared to the desert halophytes of northwest China (396.7 ± 45.4 g·kg^-1^, 28.1 ± 9.4 g·kg^-1^, and 1.85 ± 0.5 g·kg^-1^, respectively) (Wang et al., 2015), as well as global terrestrial plants (461.6 ± 72.2 g·kg^-1^, 20.1 ± 8.7 g·kg^-1^, and 1.77 ± 1.1 g·kg^-1^, respectively) (Elser et al., 2000b; Reich and Oleksyn, 2004). Despite SJ4 and SJ5 had more symbiotic species than the other three habitats—three and four species, respectively—the LOC, LTN, and LTP of these two habitats were not the greatest in comparison to the other three habitats (**Figures 3D-F**). This conclusion does not accord with the view that the more species in the community, the higher the element concentrations of plant leaves (van Ruijven and Berendse, 2005; Oemlann et al., 2007; Abbas et al., 2013). Interestingly, LTN of *T. chinensis* in SJ3 was significantly higher than that of the other four habitats, which may mainly due to nitrogen fixation of leguminous plants (*S. alopecuroidies*) promotes the N concentration of non-leguminous plants (Nyfeler et al., 2009; Zeng et al., 2010; Zhang et al., 2016). The coupling between plant-soil P often occurs at the ecosystem scale (Aerts and Chapin, 2000). The correlation between the P content of leaves and soil P of terrestrial plants in China has also supported this viewpoint. Our study adds to the previously stated perspective by demonstrating a substantial positive association between STP and LTP (*P* < 0.05) (**Figure 4**).

Forth, compared with SJ1, SJ2, and SJ3, where there are fewer species, we concluded that LOC: LTN and LOC: LTP of SJ4 and SJ5 were the highest (**Figures 3G-I**). This may be a result of increased species variety and the complementary between species occupying various resource niches, which boosts the community’s ability to use its resources effectively (Robertson et al., 2009). Furthermore, it is possible that the presence of leguminous plants (*A. sparsifolia*) has an influence on the stoichiometric ratio of the elements in leaves (Nyfeler et al., 2009; Zeng et al., 2010; Zhang et al., 2016), and more data should be acquired to validate this hypothesis. Moreover, our result is supported by earlier researches that indicated that GWD was also a source of plant nutrients in addition to soil nutrient condition could be in part explanation, given that higher nutrient availability results in higher nutrient concentrations in leaves (Aerts and Chapin, 2000; Zhang et al., 2018), therefore LOC: LTN was significantly and positively correlated with SOC: STN (*P* < 0.05), while LOC: LTP was significantly and negatively correlated with STP and GWD in our study (*P* < 0.01) (**Figure 4**). Leaf N: P measures the relative limitation of N versus P. Generally, an N: P value below 14 frequently suggests limitation in N, whereas the value above 16 frequently shows limitation in P (Aerts and Chapin, 2000). The mean LTN: LTP values in this study (16.58 ± 2.88) are higher than in others (Elser et al., 2000a; Reich and Oleksyn, 2004), probably suggesting that *T. chinensis* is relatively limited by soil P concentration.

### 4.2 Correlations of leaf functional traits

Previous empirical studies have reported that correlational constraints exist among inter-relationship traits, and a mixture of direct and indirect causal relationships are reflected between traits in different environments (Wright et al., 2005; Shipley et al., 2006; Enquist et al., 2007; Lavorel, 2013) at large spatial scales. Our local scale research revealed that leaf functional traits were functionally associated across *T. chinensis* individuals within the five different habitats.

We found that LWC was significantly negatively correlated with *δ* ^13^C (**Figure 5**), LOC: LTN and LOC: LTP, while *δ* ^13^C was significantly and positively correlated with LOC: LTN but negatively correlated with LTN. In generally, leaf functional traits don’t vary independently, their coordination indicate ecological trade-offs between acquiring and conserving resources (Yang et al., 2019; Kang et al., 2021). The correlation between LWC and *δ* ^13^C in our study is consistent with the significant negative correlation found by previous study on these two traits of major species in Xilin River Basin (Chen et al., 2007). In other words, the species with lower LWC had higher WUE and exhibited more conservative patterns of water usage. In our study,*δ* ^13^C was negatively correlated with LTN, our finding contradicts the commonly reported positive correlation between these two trait (Sparks and Ehleringer, 1997). Within a certain range, the increase of N concentration promotes photosynthesis, thereby has an impact on the increase of WUE (Hamerlynck et al., 2004; Li et al., 2022). On the contrary, studies also found that *δ* ^13^C was negatively correlated with leaf N concentration, and this was limited by season (Tsialtas et al., 2001). Prior research on trait-trait correlation has tended to concentrate on and quantify the link between SLA and N and P concentrations in leaves (Wright and Cannon, 2001; Wright et al., 2004b). Under extreme drought environment, plants generally choose the conservative strategy with lower SLA and lower N and P concentrations in leaves, and these traits are frequently positively connected (Yang et al., 2019). We found no association between these three traits, which may have been because we could only observe them for one season. Long-term research is thus required.

Given the above context, *T. chinensis* from the five habitats we investigated, with different vegetation communities, we can conclude that *T. chinensis* response to the native extremely arid and barren environments with higher LWC pair with lower *δ* ^13^C, and exhibiting lower C, N, P in leaves and their stoichiometric ratios compared with other terrestrial plant, as well as lower SLA compared to *Tamarix* growing in habitats with abundant resources. Overall, the above trait-trait combinations, suggesting that *T. chinensis* develops a suite of trait combinations mainly tends to more conservative, and *T. chinensis* seems to be a slow-investing species in these five communities. This is consistent with the theory that species grow quickly and thus grab more resources when these are abundant, but avoid mortality under resources are scarce (Russo et al., 2005; Poorter and Bongers, 2006; Wright et al., 2010). If we want to be more clearly about its trait combination and which end of LES it is in, we need to make more in-depth studies on more traits such as leaf lifespan, photosynthetic capacity per unit leaf mass, and so on.

## 5 Conclusion

Overall, there were distinctions between leaf functional traits exists within the five habitats at the individual level. Abiotic factors vitally influence leaf functional traits, of which groundwater depth and soil C: N stoichiometry are crucial. Furthermore, the presence of leguminous species, or species diversity, may explain why LOC: LTN and LOC: LTP for SJ4 and SJ5 were higher than the other communities. Leaf functional traits we selected were not independent. LWC was significantly negatively correlated with *δ* ^13^C, LOC: LTN and LOC: LTP, while *δ* ^13^C was significantly and positively correlated with LOC: LTN but negatively correlated with LTN. Moreover, *T. chinensis* develops a suite of trait combinations mainly tends to more conservative, and *T. chinensis* seems to be a slow-investing species in these five communities to response local habitats in Daliyabuyi.

## Data availability statement

The datasets presented in this article are not readily available because the participants of this study did not agree for our data to be shared publicly.

## Author contributions

YD and QS conceived the study. MT, YD, AA, FE, WH led the sample collection. MT analyzed the data. MT and YD led the writing of the manuscript. All authors contributed to the article and approved the submitted version.
